# Offending Trajectories in Violent Offenders: Criminal History and Early Life Risk Factors

**DOI:** 10.1177/0306624X221086565

**Published:** 2022-04-18

**Authors:** André Tärnhäll, Jonas Björk, Märta Wallinius, Peik Gustafsson, Björn Hofvander

**Affiliations:** 1 LU-CRED, Child and Adolescent Psychiatry, Department of Clinical Sciences Lund, Lund University, Lund, Sweden; 2 Department of Forensic Psychiatry, Region Skåne, Trelleborg, Sweden; 3 Centre of Ethics, Law and Mental Health, Department of Psychiatry and Neurochemistry, Institute of Neuroscience and Physiology, The Sahlgrenska Academy at University of Gothenburg, Gothenburg, Sweden; 4 Department of Occupational and Environmental Medicine, Lund Universisty, Lund, Sweden; 5 Research Department, Regional Forensic Psychiatric Clinic, Växjö, Sweden

**Keywords:** criminal behavior, violent offenders, longitudinal, group-based modeling, risk factors

## Abstract

The understanding of offending, and thus its possible prevention, is expanded through longitudinal studies on criminal trajectories depicting early life risk factors. This longitudinal study aimed to explore criminal trajectories, criminal histories, and early life risk factors in a cohort of violent offenders. A Swedish nationally representative cohort of male violent offenders (*n* = 266), clinically assessed while imprisoned aged 18 to 25, was followed through national registers from age 15 to 25–34. Substantial differences in criminal histories between violent offenders and a matched comparison group (*n* = 10,000) were demonstrated. Five trajectory groups were identified: four persisting and one desisting. Although differences were observed between persisting trajectory groups, a higher prevalence of early life risk factors was generally displayed compared to the desisting, especially in conduct problems and experiences of out-of-home placements. Neurocognitive ability and prevalence of ADHD and autism were similar across trajectories. Severe early life risks highlight the population’s need for early interventions.

The empirical study of offending trajectories furthers knowledge about the timing, degree, and nature of criminal behaviors at the individual level (Nagin, 2005). Earlier studies have found evidence of two to seven trajectories of antisocial, criminal, or aggressive behaviors (Jennings & Reingle, 2012; Piquero, 2008). The “age-crime curve” illustrates that criminal behaviors, on an aggregated level, begin in late childhood or early adolescence, peak in late adolescence, rapidly decrease in young adulthood, and dwindle in middle-adulthood (Farrington, 1986). Arguably (e.g., Moffitt, 1993), this age–crime relationship conceals two or more groups. Although one single trajectory has been proposed (Gottfredson & Hirschi, 1990), several developmental paths to antisocial or criminal behaviors are commonly suggested (e.g., Farrington et al., 2019; Frick & Viding, 2009; Loeber et al, 1993; Moffitt, 1993). Moffitt (1993, 2018) proposed two main developmental trajectories of antisocial behaviors: life-course persistent (LCP) and adolescence-limited (AL). While the AL group closely follows the age-crime curve, the LCP group follows a chronic antisocial course. The LCP group is marked by neuropsychological deficits associated with a myriad of risk factors unfolding in childhood, adolescence, and later, not present in the AL group. LCP individuals, more often than others, experience difficult parent–child interactions during childhood that worsen already present behavioral and temperamental issues. These risks, in turn, increase the possibility of later adverse life events. The LCP path is associated with early life risk factors (Moffitt & Caspi, 2001), and a life complicated by health problems (Odgers et al., 2007).

The described prevalence of these two offender groups in the general population varies considerably in the literature. In a review of 55 longitudinal prospective studies, based on community samples, Jolliffe et al. (2017) found a prevalence of LCP offenders ranging from 3% to 17%, and of AL offenders from 4% to 82%. For both groups, negative life events, such as substance abuse, imprisonment, victimization, or educational failures, can have ensnaring effects, binding the person to an antisocial course into adulthood with diminishing chances to change the lifestyle (Moffitt, 2018). Evidence of an offending trajectory beginning in adulthood has been reported (e.g., Eggleston & Laub, 2002). However, Moffitt et al. suggest that the proposed adult-onset group has had early life conduct problems that have gone undetected by police (Beckley et al., 2016; Moffitt, 2018). Sampson and Laub’s (2003) study of life-course offending found that, although childhood risk factors predicted levels of offending moderately well, the same childhood risk factors did not produce distinct trajectories of offender groups, in contrast to Moffitt’s (1993) typological approach. Further, they described that offending rates dwindle with age even in highly active offenders.

Studies of offender populations (e.g., Jennings & Reingle, 2012) have, in line with earlier predictions (Moffitt, 1993), supported the notion of at least one desisting and one persisting trajectory. A large body of evidence points to a disproportionally small group of offenders being responsible for the vast majority of the total burden of crime (e.g., Martinez et al., 2017). It has repeatedly been found that the group responsible for severe antisocial behaviors constitutes approximately 5% of the general population (Vaughn et al., 2011). Within offender populations, those convicted of violent crimes seem to constitute a particularly crime-prone population (Piquero et al., 2012), often accompanied by an early onset of criminal behaviors and long criminal careers (Farrington, 2019). Aggressive behaviors in the form of violent crime seem to be distinguished from other types of non-aggressive antisocial behaviors by a far more prominent genetic influence (Burt, 2009; Frisell et al., 2011). Loeber et al. (1993) proposed that violent offenders have followed a so-called overt pathway, characterized by early life minor aggression, succeeded by physical fighting early in life, preceding a longitudinal development toward violent crime. Usually, each step toward violent crime is accompanied by parallel advancement in other antisocial paths, namely, the authority conflict and covert paths (Loeber et al., 2008). This parallel advancement explains that persistent violent offenders are expected to often have committed a broad range of crimes during their criminal career (Falk et al., 2014).

In a literature review built principally on Moffitt (1993), Loeber et al. (1993), and subsequent empirical evidence, Piquero et al. (2012) present five conclusions concerning violence in criminal careers from a developmental life-course perspective: (1) most offenses during the criminal career are nonviolent, (2) evidence points toward violent offenders being frequent, rather than specialist, offenders, (3) recidivism in violence is rare, (4) recidivism in nonviolent offenses is common, and (5) nonviolent crimes usually precede violent crimes. Following these conclusions, areas of future research are proposed, including: (1) durations of criminal careers in violent offenders, (2) assessments of early life antecedents to violence, and (3) continued application of descriptive methods to understand the criminal careers of violent offenders.

Generally, relatively little is known about persistent offenders (Jolliffe et al., 2017), and less about violent offenders specifically (Piquero et al., 2012), especially highly enriched populations, such as those violent offenders who also have experienced imprisonment early in life. The population size of such highly crime-prone individuals, and to what extent they are overrepresented in criminal statistics compared to the general population, is yet unclear (Vaughn et al., 2011). Though previous studies have reported on offending trajectories in antisocial groups (Jennings & Reingle, 2012), these studies largely concern general population samples or young delinquents. Some studies have reported on offending trajectories in samples of serious offenders (Jennings & Reingle, 2012; Loeber et al., 2008), but seldom in explicitly violent offender samples with an experience of incarceration (McCuish et al., 2015), and infrequently focus on the general pattern of offending in violent offenders, as compared to specifically violent or other serious offences. Young adulthood is a crucial developmental stage in the understanding of offending progression through the life-course, but few studies report on offending throughout young adulthood (Kessler, 2020; Sampson & Laub, 2003), especially in violent offender samples. Additionally, despite its argued value (Moffitt, 2018; Moffitt & Caspi, 2001), reports of wide arrays of early life risk factors (e.g., Odgers et al., 2008) across offending trajectories in violent offenders are rare (McCuish et al., 2015). Studying early life risk factors across trajectories of general offending in samples of violent offenders can help assess whether early life risk factors help to distinguish persistent from desisting offending patterns in violent offenders (Moffitt, 1993; Sampson & Laub, 2003), as well as to further inform research about the violent offender’s criminal career (Piquero et al., 2012), and preventive interventions and treatments (Bernstein et al., 2021; Gibbon et al., 2020) in a population with most severe societal impact (Krug et al., 2002; Rivenbark et al., 2018).

The main aim of the present study was to describe the criminal activity and to explore longitudinal offending trajectories from age of criminal responsibility to adulthood in a Swedish, nationally representative cohort of males imprisoned for violent offenses when aged 18 to 25. Specifically, we aimed to: (1) compare life-course criminal convictions in the cohort to the general population, estimating the proportional size of the study population and the magnitude of its increased propensity toward crime, (2) identify offending trajectories in violent offenders and explore these through life-course criminal behaviors, assessing evidence of persistence and desistance, and (3) compare early life risk factors across trajectory groups.

## Method

### Participants and Procedure

The 266 participants were included in the Development of Aggressive Antisocial Behavior Study (DAABS), a nationally representative Swedish longitudinal closed-cohort study consisting of male violent offenders aged 18 to 25 years at inclusion. The participants were imprisoned, convicted of violent and/or “hands-on” sexual offenses, in 2010 to 2012. During this period, the participants were assessed at one of nine correctional facilities in the western region of the Swedish Prison and Probation Service. This region serves about one fifth of the Swedish prison population and runs facilities spanning all security levels. During the given period, 420 individuals were serving time for violent offenses. Forty-two were excluded, either due to lack of proficiency in the Swedish language (*n* = 23), or due to insufficient time (<4 weeks) remaining at the correctional facility for assessments to be organized (*n* = 19). Another 109 prisoners (29% of the remaining 378) declined participation. Analysis of basic demographic information revealed no significant differences in the type of index offense or age between those who declined to participate in the study and those who consented.

The baseline measures were conducted onsite by licensed clinical psychologists. The procedure consisted of an extensive semi-structured assessment, including a thorough review of available file material, a psychiatric diagnostic work-up, neuropsychological assessment, and self-rating questionnaires, following the LEAD (Longitudinal, Expert, All, Data) principles (Spitzer, 1983). Previous publications have provided comprehensive descriptions of the baseline data collection (e.g., Billstedt et al., 2017; Delfin et al., 2018; Hofvander et al., 2017; Wallinius et al., 2016).

Information from Swedish national registers was collected to supplement existing baseline data. In the current study, we employed national register-based data on life-course criminal behaviors from the National Crime Register (NCR). The data gathered from the NCR contain records of all criminal convictions at the district court level, including custodial and non-custodial convictions, from the time of criminal responsibility (15 years of age) onward. The district court is the first level instance of the general courts in Sweden. Thus, all criminal first-level convictions are captured, whilst omitting potential decisions from courts of appeal. Virtually no data are missing in the NCR (Brottsförebyggande rådet, 2018). Each conviction can involve multiple crimes and sanctions resulting from a court order, an abstention from prosecution, or a summary imposition of a fine. The NCR includes people who commit crimes under the influence of a severe mental illness and are later referred to forensic psychiatric care. Plea bargaining is not an option in Sweden, thus, there is no risk of crimes being omitted from the NCR for this reason. Swedish prison population rates are similar to those in most other Northern and Western European countries, but lower than the global average (Walmsley, 2019). Additionally, we employ data from the Longitudinal Integration Database for Health Insurance and Labor Market Studies (LISA). To account for premature deaths and migration, the Cause of Death and Migration Register were linked to available data. The register-based data include information from the earliest point of data available in the respective registers (NCR from age 15 years and LISA from 16 years) to the end of 2017. Personal identification numbers were not found for three participants, leaving a total study group of 266 participants. By the end of the follow-up period, 248 (93%) participants were alive, aged from 25 years, 5 months to 34 years, 7 months (*M* = 28.1, *SD* = 2.3).

An age- and gender-matched (all-male) comparison group consisting of 10,000 individuals from the general population was created and anonymized by Statistics Sweden. The distribution of year of birth mirrored the DAABS cohort. The comparison group was included as of 15 February 2010 (corresponding closely to the initiation of the DAABS) and followed to the end of 2017 when 9,946 (99%) were alive. Register-based information for the comparison group similar to that of the study group was collected.

The DAABS participants were given oral and written information about the study and signed informed consent. The DAABS study and the recent addition of the comparison group were approved by the Research Ethics Committee at Lund University (registration numbers 2005/698 and 2018/626, respectively). All data have been treated per ethical guidelines and the register holders’ requirements.

### Measures

#### Criminal behaviors

Complete records of criminal behaviors were gathered from the NCR. All crime categories include attempted and aggravated forms wherever applicable. Violent crime was defined as homicide, manslaughter, assault, robbery, threats, and/or violence against an officer, interference in a judicial matter, gross violation of integrity, unlawful coercion and threats, kidnapping, illegal confinement, arson, or extortion (closely following previous Swedish definitions of violent crime, see, e.g., Falk et al., 2014; Fazel et al., 2014, with minor revisions in accordance with United Nations Office on Drugs and Crime, 2015). Homicidal violence was defined as murder and voluntary or involuntary manslaughter. Aggravated violence was defined as aggravated assault, kidnapping, or aggravated robbery. Sex crimes are largely violent in nature but might be etiologically distinguished from other violent crimes (Frisell et al., 2011; Långström et al., 2015; Lussier, 2005). Thus, sex crimes were not included in the general category of violent crime and assigned their own category. Seven nonviolent crime categories were created and classified as follows: (1) theft, (2) vandalism, (3) traffic, (4) weapons-related, (5) drug-related, (6) fraud and economic offenses, and (7) other crimes (a full detailed report of crimes included in each category is available from the corresponding author upon reasonable request). A crime variety index was created, cumulatively summing the number of different categories of crime each offender had been convicted of on at least one occasion.

The main outcome measure in the analysis of offending trajectories was the total number of crimes each participant had been convicted of at each calendar age, from age of criminal responsibility (15 years) onwards. This yearly outcome measure marks the total number of crimes the participant was convicted of during the specified calendar year. Due to the relatively broad age span among the participants, the investigation of this yearly outcome measure was limited to the age span of 15 to 29 years. The outcome measure was restricted using information on immigration and premature death, as it is crucial when dealing with high rates of offending (Piquero, 2008). Thus, in the case of death or immigration after the age of criminal responsibility, the yearly outcome measure was correctly rendered a missing value rather than zero for the years in question.

The trajectory groups were labeled by their shape and level, and organized by desistance or persistence in criminal behaviors. We defined persistence through the duration of the criminal career exceeding that of the comparison group by at least 1*SD* (McGee et al., 2020). The length of a criminal career was defined as the time between the first and last criminal conviction. Desistance was operationalized through the shape and level of the trajectory group reaching, or nearly reaching, termination of the criminal career, while not fitting the criteria of persistence.

#### Early life risk factors

##### Parental and socioeconomic background

Low parental education was measured at the first available time-point available in LISA and defined as neither parent having finished high school. When information regarding only one parent’s educational level was available (*n* = 47), that represented the parental educational level. The definition of low education level among the participants was the same as that for their parents: not having completed high school. Being born outside Sweden and/or having both parents born outside Sweden were assessed through register data. We used the family’s disposable income per consumption unit (total family income/family’s total weighted consumption index) as a proxy to estimate a low income during upbringing. Utilizing the comparison group to gain information on the general population’s income levels, the lowest quartile family income per year group at age 16 (when the information at age 16 was not available, the first year of non-missing data was used), was compared with the three highest quartiles of family income within the comparison group. This information was used to create a dichotomous variable in which the value of the lowest comparison group quartile was considered to identify low family income during upbringing in the DAABS cohort (in close accordance with Fazel et al., 2014). At baseline, pedigrees charting family liability were created in collaboration with the participants (*n* = 219), from which parental criminality was established using both self-reports and data from files.

##### Adverse experiences during upbringing

This was defined as having been bullied, having been a witness or victim of repeat domestic violence, parental substance abuse, and placement in a foster home or institutional care. In Sweden, there are two reasons for placement outside the home: severe deficits in parental care or destructive individual behavior, such as delinquency. Foster home placements follow decisions by social services. Institutional care follows either a verdict from social services or, between the ages of 15 (age of criminal responsibility) and 18, a court conviction. Data were collected at baseline.

##### Neurocognition

Intellectual functioning was measured at the clinical assessment through the General Ability Index (GAI) of the Wechsler Adult Intelligence Scale—Third Edition. The GAI consists of the Verbal Comprehension Index (VCI; subtests: information, similarities, and vocabulary) and Perceptual Organization Index (POI; subtests: block design, matrix reasoning, and picture completion). In total, assessments of neurocognition were available for 261 participants.

##### Neurodevelopmental disorders

Diagnostic evaluations of neurodevelopmental disorders followed a structured interview protocol according to the Diagnostic and Statistical Manual of Mental Disorders, 4th edition, text revision (American Psychiatric Association [APA], 2000). Participants were assessed for attention deficit hyperactivity disorder (ADHD) in childhood, autism spectrum disorder (ASD), and dyslexia.

##### Conduct problems

Diagnostic information regarding the number of conduct disorder (CD) symptoms and childhood-onset CD (onset before 10 years of age) are reported. The number of CD symptoms was defined as the number of CD criteria A symptoms at 15 years of age (APA, 2000; variable properties in Supplemental Table S1). Information about the age at onset of use of alcohol, use of drugs (including cannabis), self-reported offending (variable properties in Supplemental Table S1), bullying others, and truancy was collected during the semi-structured interview at baseline. The age of onset variables were informed by all available information during the baseline interview (Wallinius et al., 2016). Agreement between official and self-reported onsets of criminal behaviors in the DAABS cohort was explored using Kendall’s coefficient of concordance, a moderate agreement between the official and self-reported onsets of criminal behaviors was found (*W* = 0.65, *p* = .007).

### Analytic Strategy

We used group-based trajectory modeling (GBTM; Nagin, 2005) to explore longitudinal offending trajectories. GBTM is a semiparametric mixed Poisson model, developed specifically to analyze longitudinal data of criminal behaviors (Nagin & Land, 1993). Its purpose is to identify groups of individuals following similar trajectories on a single outcome variable repeated over time. GBTM does not assume that the population in question is composed of a distinct number of groups. Population variability is captured by differences between groups in the shapes and levels of their trajectories. Using a polynomial link function between age and the outcome, GBTM identifies developmental trajectories, within a population, of individuals following a similar course based on actual preceding behaviors (Nagin, 2015).

In this study, the number of crimes in the DAABS cohort at each calendar age was the main outcome. The zero-inflated Poisson model was used. Following Nagin’s (2005) suggestions, we decided to set a minimum of one and a maximum of six groups allowed for the model. Exploring the groups with each (one to six) polynomial fixed to one category at a time (constant-only, linear, quadratic, and cubic), we evaluated the best fit for the current cohort. Two criteria were then applied to determine the best trajectory model: (1) The Bayesian Information Criterion (BIC) and (2) substantive significance with co-occurring conceptual clarity. A parsimonious model was favored. BIC is a formal statistical criterion that guides the researcher in determining the number of groups, rather than simply concluding the appropriate number of groups.

Mann-Whitney *U* rank-sum test, Kruskal-Wallis equality-of-populations rank test, and Pearson’s chi-square test were used to analyze criminal history and early life risk factors. Criteria for parametric tests of continuous variables were not met for any variables regarding the criminal history or most of the early life risk factors, thus, non-parametric tests were used throughout.

Sensitivity analyses using prison sentence length as a time-varying covariate to trajectory models were conducted (see Supplemental Material; Piquero et al., 2001). However, due to large degrees of uncertainty about the actual length and timing of the period of incapacitation, this covariate was not included in the main analysis. No substantial changes from the final model without time-varying covariates were found in the level, shape, or proportions assigned to each trajectory group (see Supplemental Material for further information). Further, mixed sets of polynomials were explored, but no stronger model was rendered.

The statistical software Stata (version 15) was used in all analyses. GBTM analysis was conducted with the Stata plug-in *traj* (Jones & Nagin, 2013). The level of statistical significance was set to *p* < .05.

## Results

Life-course criminal behaviors and imprisonment are presented in Table 1. Mann-Whitney *U* tests indicated that the comparison group had a significantly lower average number of crimes, convictions, and lengths of imprisonment, as well as a later age of onset and shorter criminal career duration for all variables presented in Table 1. In the comparison group, 2,371 (24%) were convicted of at least one crime during the follow-up period. In the DAABS cohort, a total of 8,728 crimes had been committed by the 266 offenders, while a total of 12,326 crimes had been committed by the 10,000-member comparison group, indicating a ratio of 26.62 (95% CI [25.90, 27.36]), comparing the average DAABS participant with the average participant from the comparison group during the follow-up period.

**Table 1.** Life-Course Criminal Behavior and Imprisonment Across Groups.

	Violent offenders						Comparison group
	Total (*n* = 266)	L-D (*n* = 83)	M-P (*n* = 91)	H-LP (*n* = 39)	H-EP (*n* = 36)	H-IP (*n* = 17)	Total (*n* = 10,000)
Crime category							
All crimes	32.81 (30.46)	8.10 (4.75)	24.48 (9.26)	55.00 (19.49)	49.72 (15.51)	111.35 (37.13)	1.23 (6.06)
Violent	7.23 (6.29)	3.10 (2.57)	6.80 (4.51)	10.21 (6.97)	11.61 (6.33)	13.53 (10.35)	0.20 (1.23)
Sex	0.29 (1.09)	0.42 (1.05)	0.37 (1.50)	0.10 (0.39)	0.03 (0.17)	0.18 (0.73)	0.01 (0.16)
Theft	4.70 (6.72)	0.90 (1.25)	3.20 (3.48)	7.56 (7.49)	9.36 (6.69)	14.88 (13.18)	0.17 (1.21)
Vandalism	0.96 (1.79)	0.43 (0.86)	0.80 (1.58)	1.18 (1.47)	1.56 (2.56)	2.65 (3.22)	0.06 (0.45)
Traffic	6.10 (10.88)	0.75 (1.44)	2.69 (3.17)	10.87 (7.46)	9.28 (9.05)	32.82 (23.06)	0.27 (2.05)
Drug-related	8.42 (10.03)	1.49 (2.05)	6.73 (5.17)	16.08 (9.01)	10.11 (5.19)	30.12 (18.11)	0.32 (1.81)
Weapons-related	2.04 (3.44)	0.28 (0.85)	1.29 (1.77)	3.23 (2.86)	3.75 (3.43)	8.35 (7.62)	0.05 (0.47)
Fraud and economic	1.64 (3.09)	0.29 (0.88)	1.53 (2.93)	3.28 (4.27)	2.36 (3.10)	3.47 (4.61)	0.05 (0.47)
Other	1.43 (3.62)	0.43 (0.72)	1.08 (1.92)	2.49 (6.74)	1.67 (1.76)	5.35 (7.36)	0.08 (0.61)
Crime variety index^a^	5.24 (1.94)	3.41 (1.53)	5.29 (1.42)	6.64 (1.06)	6.81 (1.06)	7.47 (0.87)	0.46 (1.09)
Homicidal violence (%)^b^	21 (8)	7 (8)	6 (7)	1 (3)	5 (14)	2 (12)	12 (0.1)
Aggravated violence (%)^b^	115 (43)	28 (34)	43 (47)	13 (33)	24 (67)	7 (41)	67 (0.7)
Sex (%)^b^	34 (13)	18 (22)	11 (12)	3 (8)	1 (3)	1 (6)	56 (0.6)
Conviction data							
Convictions	10.90 (7.67)	4.05 (2.21)	9.97 (3.91)	16.21 (5.94)	15.64 (5.59)	27.24 (7.69)	0.64 (2.10)
Violent convictions	3.50 (2.45)	1.77 (1.22)	3.35 (1.93)	4.56 (2.36)	5.39 (2.13)	6.24 (3.58)	0.12 (0.59)
Prison convictions	2.90 (2.32)	1.33 (0.68)	2.51 (1.49)	4.80 (2.38)	3.58 (2.25)	7.00 (2.89)	0.06 (0.50)
Sentenced prison, mths	38.82 (32.26)	28.73 (27.81)	36.04 (27.86)	43.83 (27.57)	50.99 (41.78)	65.79 (39.27)	0.52 (5.33)
Sentenced institutional care, mths	0.94 (4.14)	0.49 (3.98)	0.57 (3.88)	0.51 (1.82)	2.97 (6.19)	1.76 (3.75)	0.02 (0.49)
Criminal career							
First conviction, age	16.92 (2.12)	17.82 (2.59)	16.84 (1.92)	16.85 (1.35)	15.28 (0.51)	16.71 (2.14)	19.14 (3.71)^c^
Last conviction, age	25.60 (3.18)	23.00 (3.00)	26.74 (2.22)	26.26 (2.60)	26.58 (2.91)	28.59 (2.09)	21.79 (4.29)^c^
Criminal career (years)	8.67 (4.01)	5.18 (3.56)	9.90 (3.07)	9.41 (2.98)	11.31 (3.02)	11.88 (2.71)	2.65 (4.00)^c^

*Note*. Total mean and standard deviation in parenthesis. L-D = low-rate desisters; M-P = moderate-rate Persisters; H-LP = high-rate late-peak persisters; H-EP = high-rate early-peak persisters; H-IP = high-rate inclining persisters.

a Number of crime categories.

b *n* (*%*).

c Cases with at least one conviction (*n* = 2,371).

At the median date of baseline assessment in DAABS (30 May 2011), 1,820 (18%) had been convicted of a crime in the comparison group, and 537 (5%) had been convicted of a violent or sex crime. Of these 537 individuals only 77 (<1%) had been imprisoned before the set date. Thus, less than 1% of the 10,000 in the comparison group had experienced circumstances mirroring the inclusion criteria for the DAABS. This subgroup was responsible for 2,845 (23%) of the total 12,326 crimes in the comparison group. Comparing this subgroup of offenders (*n* = 77) in the general population group to the DAABS cohort, Mann-Whitney *U* tests revealed no overall statistically significant differences in total crime (*M* = 36.95, *SD* = 35.60 vs. *M* = 32.81, *SD* = 30.46, *p* = .64), violent crime (*M* = 7.29, *SD* = 7.03 vs. *M* = 7.23, *SD* = 6.29, *p* = .76), or sex crime (*M* = 0.16, *SD* = 0.49 vs. *M* = 0.29, *SD* = 1.09, *p* = .74). Comparisons of the DAABS cohort to violent offenders in the general population that had not experienced imprisonment revealed statistically significant differences in total crime (*M* = 32.81, *SD* = 30.46 vs. *M* = 8.62, *SD* = 13.36, *p* = .001) with a ratio of 3.81 (95% CI [3.67, 3.95]), violent crime (*M* = 7.23, *SD* = 6.29 vs. *M* = 2.52, *SD* = 2.98, *p* = .001), and sex crime (*M* = 0.29, *SD* = 1.09 vs. *M* = 0.08, *SD* = 0.38, *p* = .001).

### Longitudinal Offending Trajectories in Violent Offenders

A total of 3,564 time points were identified in the DAABS cohort (*n* = 266), giving an average number of 13.4 time points per study participant between 15 and 29 years of age. Cubic polynomials demonstrated the best fit in the current group and were applied in the models discussed below. BIC values improved with the addition of every new group, consequently, the six-group model maximized the BIC score (see Supplemental Table S2). However, the six-group model produced one trajectory encompassing a very small group of—briefly—extremely active criminal offenders, distorting the conceptually clear trajectory tendencies found in each preceding model. The more parsimonious four-group model depicted the following groups: Low-rate Desisters, Moderate-rate Persisters, High-rate Early-peak Persisters, and High-rate Inclining Persisters. However, the four-group model concealed a trajectory group of High-rate Late-peak Persisters as was evident in the five-group model. Thus, we decided upon the five-group model as the final trajectory model.

**Figure 1.**
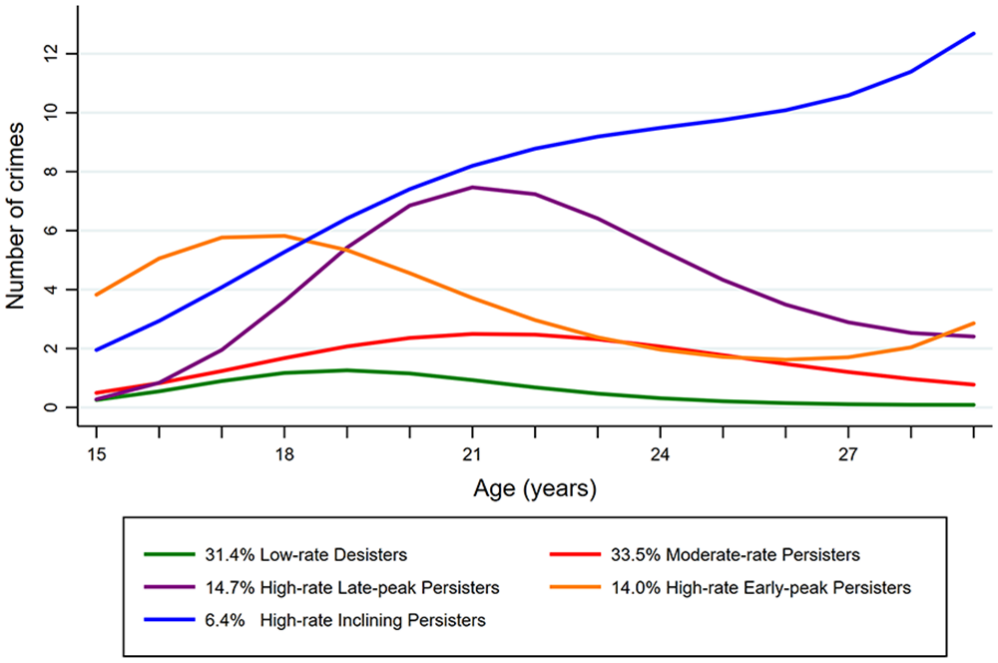
Trajectories of criminal behaviors.

Figure 1 depicts the final five-group cubic model. Roughly one third (31.4%; *n* = 83) of the cohort followed a trajectory characterized by a relatively low number of criminal offenses and a desisting offending pattern. The majority of crimes in this group were concentrated at 18 to 20 years of age, peaking at age 18 (*M* = 1.57, *SD* = 2.79). In the late twenties, the average yearly crime rate varied from 0.22 (*SD* = 0.75) at age 26 to 0.06 (*SD* = 0.24) at age 29. This first trajectory group was labeled Low-rate Desisters (L-D). Another third of the cohort, 33.5% (*n* = 91), followed a trajectory characterized by an early incline in criminal behaviors mirroring L-D, albeit with a continued incline, peaking at age 21 (*M* = 2.68, *SD* = 3.48). This trajectory group was marked by a subsequent slow deceleration, with non-trivial offending rates maintained throughout the studied period. Correspondingly, owing to the persistence across the study period and relatively moderate rates of offending, this second trajectory group was labeled Moderate-rate Persisters (M-P). A smaller group consisting of 14.7% (*n* = 39) of the cohort followed a trajectory characterized by high rates of offending at 18 to 25 years of age, also peaking at age 21 (*M* = 9.33, *SD* = 6.53), persisting throughout the follow-up period. Accordingly, this third trajectory group was labeled High-rate Late-peak Persisters (H-LP). A similar proportion, 14.0% (*n* = 36) of the DAABS cohort followed a trajectory characterized by strikingly high rates of offending after the age of criminal responsibility, peaking at age 16 (*M* = 5.81, *SD* = 5.47), followed by a slight decline in offending, yet persisting during the entirety of the study period. Thus, this fourth trajectory group was denoted High-rate Early-peak Persisters (H-EP). The smallest trajectory group, 6.4% (*n* = 17), was characterized by inclining rates of criminal behaviors during the study period, peaking at age 28 (*M* = 15.46, *SD* = 11.68) at the highest rates of any group at any age found in the cohort. This fifth and last trajectory group was labeled High-rate Inclining Persisters (H-IP).

Model diagnostic criteria (see Supplemental Table S3; Nagin, 2005), demonstrated that the average posterior probabilities of group membership ranged from 95.3% to 98.9%, by far exceeding the threshold of 70%. Odds of correct classification surpassed the suggested odds ratio of 5, and the estimated probability of the trajectory groups was strictly parallel to the proportion of the actual assignment to all five trajectory groups. In sum, the diagnostic evaluation indicated a model with high assignment accuracy.

Criminal behaviors and imprisonment across trajectory groups are also presented in Table 1. Kruskal-Wallis equality-of-populations rank test and Pearson’s chi-square test, in continuous and binary variables, respectively, indicated that the null hypothesis could be rejected in all variables across the five trajectory groups. The duration of the trajectory group average criminal career exceeded that of the comparison group’s by more than 1 *SD* (*M* = 2.65, *SD* = 4.00) in the M-P, H-LP, H-EP, and H-IP trajectory groups, thus filling the study’s definition of persisting in offending (*n* = 183, 68.8%). The L-D trajectory group (*n* = 83, 31.2%) did not meet this criterion.

### Early Life Risk Factors Across Trajectory Groups of Violent Offenders

Early life risk factors of antisocial and criminal behaviors are reported across trajectory groups in Table 2. Parental criminal history, low participant education, placement in a foster home and institutional care, number of CD symptoms, truancy, and age of onset of alcohol use, drug use, and self-reported criminal offending varied between the five trajectory groups on a statistically significant level. Generally, the L-D trajectory group differed from the persistent trajectory groups through a lower level of early life risk factors.

**Table 2.** Early Life Risk Factors Across Trajectory Groups (in % If Not Otherwise Indicated).

	Trajectory groups					*p* ^a^
	L-D (*n* = 83)	M-P (*n* = 91)	H-LP (*n* = 39)	H-EP (*n* = 36)	H-IP (*n* = 17)	
	%					
Parental and socioeconomic background						
Parental criminal history^b^	16	24	23	32	60	.009*
Low parental education	17	19	21	28	41	.18
Parents born outside Sweden	46	41	46	50	29	.63
Low family income	51	48	62	69	41	.14
Low education	64	73	97	94	76	.001*
Born outside of Sweden	34	23	21	28	24	.47
Adverse experiences						
Witness domestic violence	25	42	36	31	24	.18
Victim domestic violence	37	48	39	39	29	.47
Parental substance abuse	36	40	38	47	65	.23
Placement in foster home	13	31	28	33	35	.04*
Placement in institutional care	12	33	54	86	59	.001*
Bullied by others	31	23	24	17	19	.46
Neurocognition						
General ability index	95^c^	94^c^	92^c^	94^c^	91^c^	.72
Verbal comprehension index	91^c^	92^c^	91^c^	90^c^	89^c^	.77
Perceptual organization index	101^c^	98^c^	95^c^	102^c^	95^c^	.16
Neurodevelopmental disorders						
ADHD	59	62	66	77	59	.40
Autism spectrum disorder	13	9	8	8	6	.78
Dyslexia	16	27	16	17	19	.38
Conduct problems						
Conduct disorder symptoms	4.0^d^	5.5^d^	6.3^d^	7.6^d^	6.4^d^	.001*
Childhood-onset conduct disorder	19	24	29	44	35	.06
Bullied others	40	47	47	56	35	.50
Truancy	80	92	97	100	88	.004*
Onset alcohol use (years)	14.7	14.1	13.7	13.6	13.4	.02*
Onset drug use (years)	15.6	14.3	14.5	13.3	14.1	.006*
Onset criminal offending (years)	14.5	12.8	13.4	11.8	13.4	.04*

*Note*. L-D = low-rate desisters; M-P = moderate-rate persisters; H-LP = high-rate late-peak persisters; H-EP = high-rate early-peak persisters; H-IP = high-rate inclining persisters.

a *χ*^2^ test.

b *n* = 219.

c IQ-score.

d Total number.

* Significant (*p* < .05).

## Discussion

The main aim of the present study was to depict criminal convictions and explore longitudinal offending trajectories from age of criminal responsibility to adulthood in a Swedish nationally representative cohort of males imprisoned for violent offenses when aged 18 to 25.

First, we compared life-course criminal behaviors between the DAABS cohort and a matched comparison group from the general population. As expected, descriptive data showed substantial discrepancies between the groups. The DAABS cohort was distinguished by much higher rates of offending in all measured regards and had committed approximately 27 times (95% CI [25.90, 27.36]) as many crimes as the comparison group. Interestingly, the cohort had also committed almost four times (95% CI [3.67, 3.95]) as many crimes compared to violent offenders in the comparison group without experience of imprisonment in young adulthood. A small group of less than 1% (*n* = 77) emerged in the comparison group, who, similar to the DAABS cohort, had committed violent or sexual crime leading to imprisonment at a similar age. This subgroup presented a criminal history analogous to that of the DAABS cohort, indicating that the DAABS cohort, as it appeared at the time of inclusion, was representative of less than 1% of the young adult male population. In sum, this further corroborates that a disproportionally small group is responsible for much of the total burden of crime (e.g., Falk et al., 2014; Martinez et al., 2017, Vaughn et al., 2011), and portrays violent offenders who have been incarcerated in young adulthood as a more criminally enriched population than young violent offenders without such an experience.

Second, we identified five offending trajectory groups, distinct in shape and level. Evidence was found for pathways of desistance as well as persistence. One (L-D) of the five trajectories, representing roughly a third of the cohort, followed a desisting trajectory. Four trajectory groups (M-P, H-LP, H-EP, and H-IP) persisted in offending from adolescence, through young adulthood, into adulthood, clearly representing an LCP path (Moffitt, 1993). Although the M-P trajectory group is relatively moderate in offending in the current cohort, its criminal career resembles the characteristics of LCP offenders as described in previous prospective studies (Jolliffe et al., 2017). The majority of offenders persisted in offending throughout the follow-up period. Consequently, imprisonment due to violent crimes in young adulthood is generally a marker of a long criminal career with an early onset of criminal behaviors. As expected, and in accordance with Sampson and Laub’s (2003) findings, most trajectory groups displayed a reduced yearly offending rate compared to their peak rate. However, the H-IP trajectory group did not, mirroring Sampson and Laub’s (2003) High-rate chronic trajectory group, displaying increasing rates of offending even at age 29. If the H-IP trajectory group continues to mirror the High-rate chronics (Sampson & Laub, 2003), this group is not expected to display decreased crime rate until middle adulthood. Although the remaining persistent trajectory groups will likely persist in criminal behavior, a continued crime deceleration is expected. All trajectory groups displayed versatile patterns of offending, in line with the conclusions of Piquero et al. (2012). In other words, overt antisocial behaviors, such as violent crimes, were largely accompanied by other types of antisocial criminal behaviors (Loeber et al., 1993). As expected, in this cohort of violent offenders, (Loeber et al., 1993; Piquero et al., 2012), the results suggested that the persistent trajectory groups displayed higher levels of crime variety (*M* = 5.29–7.47) and a lower proportion of violent offenses (13%–29%) compared to the desisting trajectory group (*M* = 3.41, 41%), thus indicating a wider range of criminal behaviors and advancement in several developmental paths of antisocial behaviors. Further considering the conclusions of Piquero et al. (2012), this study offers evidence in favor of nonviolent crimes being more common than violent crimes among violent offenders, but in contrast, total recidivism was found to be common in both violent and nonviolent crimes, and violent recidivism was particularly frequent in the persisting trajectory groups. In the total cohort, the average criminal career length was nearly 9 years, reproducing evidence of the long criminal careers of violent offenders (Piquero et al., 2012). The four persisting trajectory groups had registered criminal careers averaging from 9 to 12 years, out of the total average follow-up period of 13 years, underscoring a remarkably early onset of registered criminal careers and little to no indication of desistance. The average duration of a criminal career in the L-D trajectory group was 5 years, longer than most studies report in AL-groups (Jolliffe et al., 2017).

Finally, we explored early life risk factors across trajectory groups. In general, the prevalence of the analyzed risk factors was substantial in the total DAABS cohort, surpassing that of the LCP-group reported by Odgers et al. (2008). Although we observed differences between persisting trajectory groups, the results reveal a pattern of early life risk factors generally distinguishing the persisting trajectory groups from the desisting. The clearest distinctions between the desisting and persisting trajectory groups were those regarding early life conduct problems and out-of-home placements. The desisting trajectory group presented on average 4.0 CD symptoms, while the persisting trajectory group exhibited 5.5 to 7.6 symptoms. This falls in with previous research showing that the total burden of early life antisocial behaviors often has serious implications on future development by being related to adverse outcomes in young adulthood and onwards (Lichtenstein et al., 2019; Loeber et al., 1993; Moffitt, 1993; Odgers et al., 2007), underscoring the importance of monitoring such behavioral displays in childhood and adolescence. The number of participants who had experienced placements in a foster home (13%–35%) and/or institutional care (12%–86%) was remarkably elevated, especially in the persisting trajectory groups, compared with the Swedish average of 3% to 4% with such experiences (Statens beredning för medicinsk och social utvärdering, 2017). Parental criminal history and substance abuse, low participant education, early age at onset of alcohol and drug use, and self-reported criminal offending were other factors that, on differing levels, were found to be more prevalent in the persisting trajectory groups compared to the desisting. Neither being a witness or victim of domestic violence, nor parental substance abuse varied between the trajectory groups, nevertheless, the overall levels of adverse experiences were alarming. Although, with some notable differences, we present evidence pointing in the direction of Moffitt’s (2018; Moffitt & Caspi, 2001) conclusion, indicating that early life risk factors do indeed aid the understanding of the development of persistent offending patterns, currently in a sample of young adult violent offenders sentenced to imprisonment. Early life risk factors distinguished trajectory groups more clearly in the current study compared to the low violence/low non-violence versus other trajectory groups in McCuish et al. (2015) study of violent offenders. Surprisingly, a childhood-onset of CD symptoms (before age 10) did not distinguish the trajectory groups on a statistically significant level (*p* = .06). The lack of a statistical significance should not be deemed an argument against the utility of the childhood-onset CD specifier (APA, 2013; Lahey et al., 1998). Rather, we observed childhood-onset CD across all trajectory groups in the current cohort, and with indications of it being more common in the persisting trajectory groups.

Levels of neurodevelopmental disorders were strikingly elevated compared to those expected in the general population (Polanczyk et al., 2015). However, the prevalence of ADHD, ASD, or dyslexia did not vary significantly across the trajectory groups, nor did the measured neurocognitive ability. ADHD and its connection to persistent offending have long been discussed. However, our results support the early findings of Lilienfeld and Waldman (1990), suggesting that childhood ADHD on its own does not predict persistent criminal behaviors in the absence of other information regarding CD or antisocial personality disorder. Further, ADHD is likely too common in the cohort to meaningfully distinguish trajectory paths. The statistical prevalence of ASD across trajectory groups did not differ significantly, further emphasizing that individuals with ASD in the current cohort are similar to those without ASD in their criminal histories (Hofvander et al., 2019). Intriguingly, all trajectory groups indicatively seemed to display, on average, lower results on the Verbal Comprehension Index compared to the Perceptual Organization Index (Moffitt, 2018).

From a clinical perspective, the characteristics of the L-D trajectory group are of certain interest. It had the oldest age at first conviction, onset of alcohol, and drug use, as well as a higher rate of sex crimes than any other group. In general, the L-D trajectory group evinced somewhat higher psychosocial stability and fewer childhood adversities compared to the other trajectory groups, except having been bullied, where they demonstrated the highest rates, as well as an elevated rate of ASD. Taken together, the clinical picture of the L-D trajectory group is not in line with the “typical violent offender,” but rather constitutes a group in which many participants might be perceived as socially atypical, a finding that might have been more evident in a larger cohort. Further, the H-IP trajectory group displayed elevated levels of parental criminal history, in combination with a tendency of low parental educational attainment, and a high prevalence of parental substance abuse, tentatively indicating an aggregated intergenerational transmission of psychosocial problems, including criminal behaviors. While the H-EP trajectory group was characterized by placements in institutional care, high levels of ADHD, childhood-onset CD, number of CD symptoms, and the earliest onset of alcohol, and drug use, as well as self-reported criminal offending, in combination with low educational attainment.

The study had several limitations. First, while it is common to employ official records in the study of criminal behavior, large discrepancies regarding frequency have been reported between official and self-reported criminal behavior (consider Moffitt et al., 2002), though, other reports have described a moderate to strong concordance between official conviction records and self-report (Fontaine et al., 2014; Piquero et al., 2014). Although focusing on official criminal behaviors, both self-reported and official conviction onset were reported in this study, and a moderate degree of concordance was found between the two. Second, the prospective follow-up period does not measure the full length of criminal careers. Thus, the criminal career characteristics reported here are not final figures but rather a description of criminal behaviors during the most criminally active developmental periods. Consequently, an extended follow-up period could have revealed that individuals, here assigned to the desisting trajectory group, actually persisted in crime. Third, the sample size brought statistical uncertainty, especially in the analysis of trajectory groups. It is unlikely that another sample of the exact same population would yield the exact same trajectory assignments found in the current sample (Jennings & Reingle, 2012). However, in-depth data, as offered in this study, are difficult to gather in larger studies. Fourth, this was not a test of taxonomic theories. Rather, we applied a descriptive statistical method, which yielded approximate results that can be understood in light of theories about the developmental pathways of criminal behaviors (Moffitt, 1993; Nagin, 2015). Fifth, in this study, risk factors were used to describe and explore the identified trajectory groups rather than to explain causal relationships, thus, no such analyses were performed. Alas, identified differences could disappear in multivariable analyses. Sixth, the cohort consisted solely of male offenders. The recruitment area operates only one facility for women and the number of women serving time for a violent offense at the time of inclusion was considered too small for meaningful statistical analysis. Since most convicted offenders are men (Walmsley, 2019), and the occurrence of serious offending is vastly reduced in women compared with men (DeLisi & Piquero, 2011), we still consider it important to study populations of male offenders.

Future research should aim to continue the exploration of heterogeneity in large cohorts of young violent offenders with an experience of incarceration. Such research could benefit from the general population comparisons presented here. Associated health outcomes such as psychopathology and healthcare utilization over the life course and across trajectory groups are of particular interest, especially when studied in multivariable models. Hence, such research efforts could help further understanding of this costly group and inform both preventive interventions and offender rehabilitation. In efforts to advance knowledge regarding criminal career duration in violent offenders (Piquero et al., 2012), and to decrease the definitional muddle regarding persistence and adjunct constructs, we argue that persistence concerns the duration of criminal behavior and should be operationalized through criminal career duration as in the current study (McGee et al., 2020).

The high levels of early life risk factors in the cohort indicate that the population requires preventive interventions at a young age to stop the subsequent development of criminal behaviors, and the associated health and societal risks. In accordance with previous research (Odgers et al., 2008), we have shown that individuals with, especially, an early onset and heavy burden of conduct problems, but also several other early life risk factors, seem to be at the highest risk of developing a persistent offending pattern, and arguably, are in the greatest need of early life interventions.

## Supplemental Material

sj-docx-1-ijo-10.1177_0306624X221086565 – Supplemental material for Offending Trajectories in Violent Offenders: Criminal History and Early Life Risk Factors Click here for additional data file. Supplemental material, sj-docx-1-ijo-10.1177_0306624X221086565 for Offending Trajectories in Violent Offenders: Criminal History and Early Life Risk Factors by André Tärnhäll, Jonas Björk, Märta Wallinius, Peik Gustafsson and Björn Hofvander in International Journal of Offender Therapy and Comparative Criminology
