# Supplementary material for: Offending Trajectories in Violent Offenders: Criminal History and Early Life Risk Factors
Source: Int J Offender Ther Comp Criminol. 2022 Apr 18;67(2-3):270–90. doi: 10.1177/0306624X221086565 (PMC9806473; doi:10.1177/0306624X221086565)
Supplement: sj-docx-1-ijo-10.1177_0306624X221086565 – Supplemental material for Offending Trajectories in Violent Offenders: Criminal History and Early Life Risk Factors [file sj-docx-1-ijo-10.1177_0306624X221086565.docx]

**Supplementary Material**

**Sensitivity Analysis Including Prison Sentence Length in Analyses of Offending Trajectories**

**Method.** For sensitivity purposes, analysis models of 1- to 6-group cubic models were complemented by adding a function allowing a time-varying covariate of prison sentence length to fluctuate freely across the time points (Nagin, 2005; Nagin et al., 2003; Piquero et al., 2001). Due to uncertainty about the actual length and timing of the period of incapacitation, this covariate was not included in the main analysis. Reasons for uncertainty were due to parole, actual date of incarceration (compared to date of conviction), and potential court appeals, not being available to us in the National Crime Register.

The here applied covariate of prison sentence lengthwas instead adjusted for parole by assuming the general Swedish parole standards. The general rule states that if an offender is sentenced to prison for a period longer than one month, parole is generally given after two-thirds of the sentence has been served, assuming no extraordinary reasons are presented in opposition. The final order of the court, which commonly marks the start of sentenced incarceration, falls three weeks after the date of conviction in district court. However, detention is regular in some cases where the defendant is suspected, on reasonable or probable grounds, of the crime or is awaiting the final order of the court. This time in remand is deducted from the subsequent prison sentence. Bail is not granted in Sweden; however, a felon can deduct time in remand from a consecutive prison sentence. The covariate uses the date of court conviction to estimate the starting point of time in incarceration, estimating the proportion of each calendar year the felon is expected to have been incarcerated from the starting date. The covariate ranges from zero to one, where zero assumes the served to time in prison that calendar year and one assumes a full calendar year of imprisonment.

Models with and without time-varying covariates cannot be compared as in an adjusted and unadjusted regression model because the covariate affect not only the model shape, but—importantly—also the trajectory assignment. With this in mind, comparing the different models in this study can still not only be of interest, but also add value, since many studies report crime rates without considering incarceration time, which complicates between-study comparisons.

**Results**. None of the six models analyzed for sensitivity purposes, and presented in Supplementary figure, substantially altered the proportions assigned to each trajectory, nor did any considerably affect the shape or level of the trajectories. Similar to models without time-varying covariates, the 5-group cubic model with prison sentence length as time-varying covariates was considered the best fit in the current cohort (BIC = −9500.95). Diagnostic evaluations indicated a high assignment accuracy. The five trajectory groups with the time-varying covariates were similar in shape, level, and size to the model that did not include the time-varying covariates, although prison sentence length had a statistically significant positive effect on the majority of trajectory groups, varying in effect sizes.

***
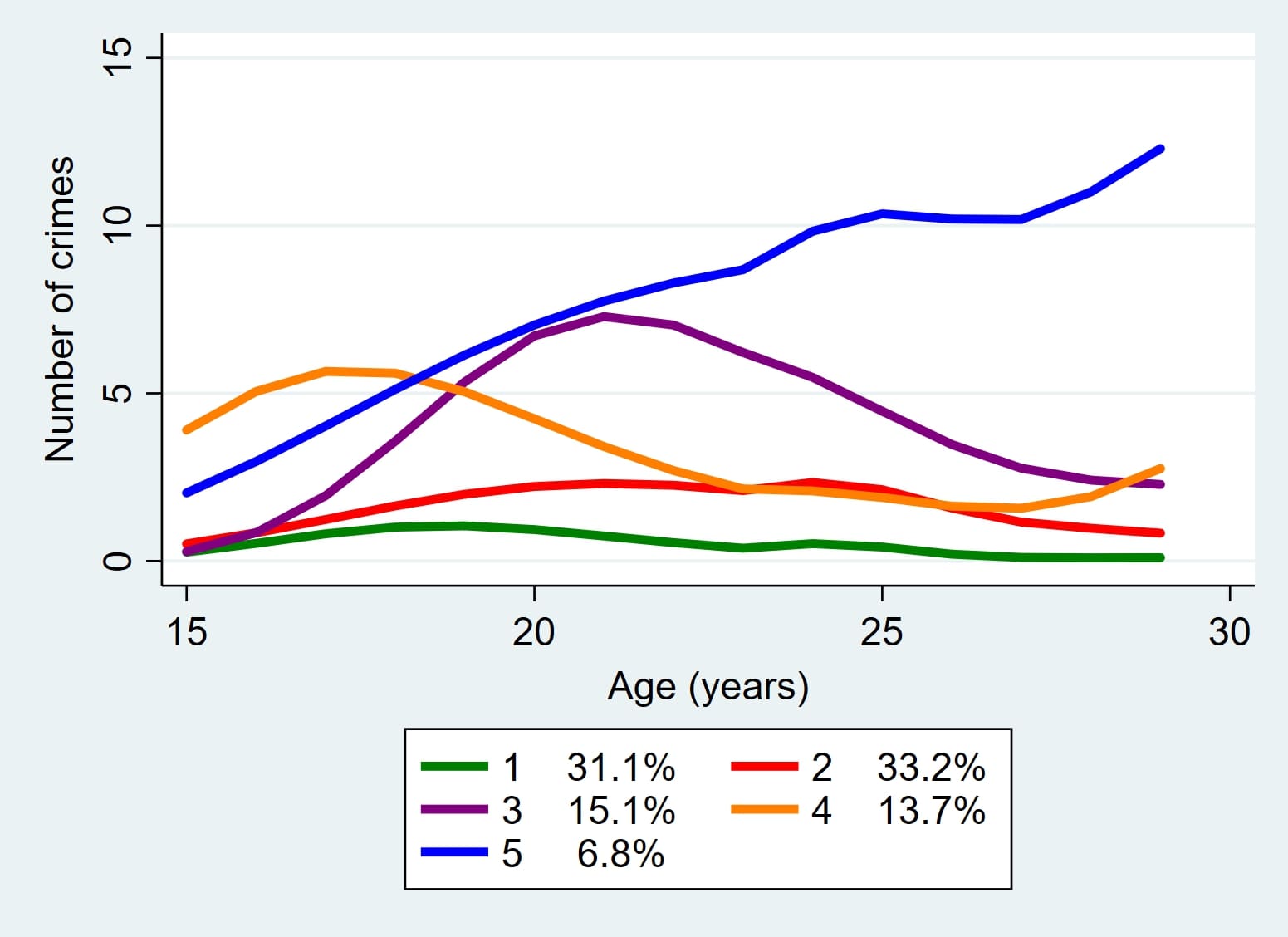
***

**Supplementary figure.** *Trajectories of criminal behaviors with prison sentence length as time-varying covariates.*

**Table S1.** Properties of risk factors.

| Risk factor | *n* | *M* | *SD* | range |
| --- | --- | --- | --- | --- |
| Onset alcohol use, yrs | 246^a^ | 14.1 | 2.31 | 8–22 |
| Onset drug use, yrs | 222^a^ | 14.5 | 2.55 | 4–22 |
| Onset criminal offending, yrs | 263 | 13.3 | 4.04 | 5–24 |
| Conduct disorder symptoms | 263 | 5.5 | 3.40 | 0–13 |

*Note*: ^a^Missing data largely due to information of lack of onset.

**Table S2.** Tabulated Bayesian Information Criterion (BIC) and 2log_e_(B_10_) in cubic models

| Number of groups | BIC | Null model | *2log_e_(B_10_)* |
| --- | --- | --- | --- |
| 1 | −12178.07 |  |  |
| 2 | −10335.49 | 1 | 3685.16 |
| 3 | −9882.31 | 2 | 906.36 |
| 4 | −9668.84 | 3 | 426.94 |
| 5 | −9500.95 | 4 | 335.78 |
| 6 | −9472.87 | 5 | 56.16 |

*Note:* The number of groups tested in the null hypothesis is denoted in the null model column. When comparing models with different numbers of groups 2log_e_(B_10_) is used, representing approximately 2(∆BIC). For a description of the method see Jones, Nagin & Roeder (2001). ∆BIC is calculated by subtracting the BIC value of the simpler model from the more complex model.

**Table S3.** Diagnostics of assignment accuracy of the final five-group cubic model

| Group | *n* | APP | OCC | Estimated % | Assigned % |
| --- | --- | --- | --- | --- | --- |
| L-D | 83 | 97.7 | 91.8 | 31.4 | 31.2 |
| M-P | 91 | 95.3 | 38.9 | 33.5 | 34.2 |
| H-LP | 39 | 97.6 | 233.6 | 14.7 | 14.7 |
| H-EP | 36 | 98.9 | 582.4 | 14.0 | 13.5 |
| H-IP | 17 | 97.3 | 522.8 | 6.4 | 6.4 |

*Note:* APP: average posterior probability; OCC: odds of correct classification

L-D: low-rate desisters; M-P: moderate-rate persisters; H-LP: high-rate late-peak persisters; H-EP: high-rate early-peak persisters; H-IP: high-rate inclining persisters
